# Multimodal Treatment of Pediatric Ruptured Brain Arteriovenous Malformations: A Single-Center Study

**DOI:** 10.3390/children8030215

**Published:** 2021-03-11

**Authors:** Lukasz Antkowiak, Monika Putz, Marta Rogalska, Marek Mandera

**Affiliations:** 1 Department of Pediatric Neurosurgery, Medical University of Silesia, 40-752 Katowice, Poland; monikaputz1977@gmail.com (M.P.); mmandera@sum.edu.pl (M.M.); 2 Faculty of Medicine, Medical University of Warsaw, 02-091 Warsaw, Poland; rogalska_marta@wp.pl

**Keywords:** arteriovenous malformations, intracerebral hemorrhage, radiosurgery, embolization, clinical outcomes

## Abstract

Bleeding from ruptured brain arteriovenous malformations (bAVMs) represents the most prevalent cause of pediatric intracranial hemorrhage, being also the most common initial bAVM manifestation. A therapeutic approach in these patients should aim at preventing rebleeding and associated significant morbidity and mortality. The purpose of this study was to determine the clinical outcomes of pediatric patients who initially presented at our institution with ruptured bAVMs and to review our experience with a multimodality approach in the management of pediatric ruptured bAVMs. We retrospectively reviewed pediatric patients’ medical records with ruptured bAVMs who underwent interventional treatment (microsurgery, embolization, or radiosurgery; solely or in combination) at our institution between 2011 and 2020. We identified 22 patients. There was no intraoperative and postoperative intervention-related mortality. Neither procedure-related complications nor rebleeding were observed after interventional treatment. Modified Rankin Scale (mRS) assessment at discharge revealed 19 patients (86.4%) with favorable outcomes (mRS 0–2) and 3 patients (13.6%) classified as disabled (mRS 3). Microsurgery ensured the complete obliteration in all patients whose postoperative digital subtraction angiography (DSA) was available. Management of high-grade bAVMs with radiosurgery or embolization can provide satisfactory outcomes without a high disability risk.

## 1. Introduction

Brain arteriovenous malformations (bAVMs) are intracranial vascular lesions characterized by abnormal connections between the arterial and venous systems without an interposed capillary bed.

Pediatric bAVMs constitute merely 12–18% of all diagnosed bAVMs [1]. Initial presentation with bAVM rupture occurs more frequently in the pediatric population than in the adult population and accounts for 58–77% of admissions [2]. Although spontaneous pediatric intracerebral hemorrhage has an annual incidence of 1.4 per 100,000 person-years [3], it carries a risk of severe permanent neurological deficits occurring in 20–40% of patients and significant mortality in up to 25% of affected individuals [4,5]. Ruptured bAVMs account for 30–50% of intracranial bleeding in the pediatric population and are the most common cause of childhood hemorrhagic stroke [1].

Multiple factors, such as diffuse and small nidus, deep and infratentorial location, deep venous drainage, single draining vein, single feeding artery, and high Spetzler-Martin grade were identified as positive predictors for the hemorrhagic presentation of bAVM in children [6,7]. Nevertheless, a prior history of ruptured bAVM remains the strongest independent predictor of re-hemorrhage [8].

A therapeutic approach in patients presenting with ruptured bAVM should prevent further rebleeding and associated significant morbidity and mortality.

Current therapeutic approaches for pediatric ruptured bAVMs include open microsurgery, endovascular embolization, stereotactic radiosurgery (SRS), applied solitary, or as a multimodal treatment strategy. In 1986, Spetzler and Martin developed a bAVM grading scale, which, based on the bAVM radiological features, can predict the surgery-related risk [9]. Accordingly, bAVMs are divided into low-grade (Spetzler-Martin grades I–III) and high-grade bAVMs (Spetzler-Martin grades IV–V). Low-grade bAVMs are perceived as suitable candidates for microsurgical resection, while high-grade ones carry a significant risk of surgery-related morbidity, and they need complex therapy.

In the event of a patient’s unsuitability for microsurgical resection, stereotactic radiosurgery—with or without prior partial bAVM embolization—might facilitate eventual bAVM resection or offer complete bAVM obliteration [10,11]. Notably, it can take up to several years until complete bAVM obliteration is achieved, and the risk of rebleeding persists in that period.

The purpose of this study was to determine the clinical outcomes of pediatric patients who initially presented at our institution with ruptured bAVMs and to review our experience with a multimodal approach in the management of pediatric ruptured bAVMs.

## 2. Materials and Methods

Following institutional review board approval, we reviewed the medical records of pediatric (<18 years of age at presentation) patients with bAVM who underwent interventional treatment in the Department of Pediatric Neurosurgery, Medical University of Silesia in Katowice between 2011 and 2020. The following inclusion criteria were established: (1) radiologically confirmed ruptured bAVM; (2) availability of detailed pretreatment angiographic data; (3) complete clinical preoperative and postoperative data; (4) surgery, embolization, radiosurgery, or a combination of the above.

The clinical data analysis included patient demographics (sex, age) and detailed symptomatic presentation (headaches, seizures, neurologic deficits). Modified Rankin Scale (mRS) scores and Hunt-Hess scale (HH) scores at admission were calculated retrospectively for all patients. Following pre-intervention digital subtraction angiography (DSA) imaging, Spetzler-Martin (SM) scores, and supplementary grading scale (Supp-SM) scores were calculated according to the following components: bAVM size, deep or superficial venous drainage, localization of the malformation, age, and diffuse or compact bAVM character. Additionally, bAVM’s detailed location and laterality were assessed. Malformation located subcortically, in the brainstem, thalamus, or basal ganglia were described as deep malformations, whereas superficial location indicated lesion involving the cerebral cortex. Imaging studies and medical records were screened for the presence of intracerebral, intraventricular, or subarachnoid hemorrhage.

In each case, the decision regarding patients’ qualification for surgery, embolization, radiotherapy, or combined therapy was made by an interdisciplinary team consisting of a neurosurgeon, neuroradiologist, and, when necessary, a radiotherapist. For patients undergoing transarterial embolization, the Onyx embolic was applied. Patients undergoing radiosurgery were transferred to the National Oncology Institute in Gliwice. The dosage of applied radiation ranged from 16 to 22 Gy.

Patients’ clinical postoperative data were carefully reviewed for the identification of any complications. A modified Rankin Scale was applied to retrospectively evaluate clinical outcomes at discharge from the hospital.

Scores ranging from 0 to 2 indicated good clinical outcomes, while those patients who received a score of 3–5 were qualified as disabled. Control DSA, performed in each patient within three months after definitive bAVM treatment, enabled one to assess the completeness of bAVM obliteration.

## 3. Results

### 3.1. Clinical Presentation

Between 2011 and 2020, 22 pediatric patients with ruptured bAVMs treated at our institution were identified. The mean age of the study cohort was 11.9 years, ranging from 2 to 17. There was an equal sex distribution (11:11). Detailed patient clinical data are presented in Table 1. Initially, 8 patients (36.4%) presented with neurologic deficit in the form of hemiparesis in 7 (31.8%) patients and hemianopsia in 1 patient (4.6%). Baseline mRS scores were as follows: 0, 13.6%; 1, 27.3%; 2, 13.6%; 3, 9.1%; 4, 9.1%; 5, 27.3%. 

### 3.2. Radiological Presentation

Eighteen bAVMs (81.8%) were located superficially, including 6 (27.3%) in the frontal lobe, 5 (22.7%) in the parietal lobe, 4 (18.2%) in the occipital lobe, 2 (9.1%) in the temporal lobe, and 1 (4.6%) in the frontoparietal area. The remaining 4 lesions (18.2%) involved deep subcortical structures. Malformation characteristics are presented in Table 2. Spetzler-Martin grades were as follows: I, 41%; II, 27%; III, 14%; and IV, 18%.

Lesions were additionally divided into low-grade bAVMs (SM I–III) and high-grade bAVMs (SM IV–V). Consequently, there were 18 (81.8%) low-grade and 4 (18.2%) high-grade bAVMs. Solitary intraparenchymal hemorrhage was present in 11 patients (50%), while intraventricular bleeding was present in 2 patients (9.1%). Both intraparenchymal and intraventricular hemorrhages were observed initially in 8 patients (36.4%). Only 1 patient (4.6%) presented with extensive intracerebral, intraventricular, and subarachnoid bleeding.

### 3.3. Outcomes

**Figure 1:**
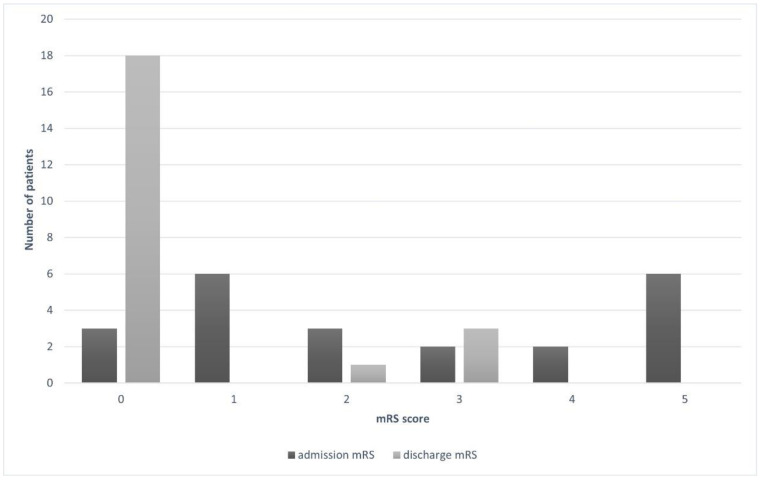
Relationship between mRS scores at admission and discharge from the hospital.

There was no intraoperative and postoperative mortality. No patient experienced rebleeding during the entire observation period. Among 8 patients who initially presented with focal neurologic deficit, 4 experienced complete physical improvement. Hemiparesis persisted in 3 patients, while the remaining 1 improved significantly. Consequently, 3 patients (13.6%) received 3 points in the mRS at discharge and were classified as disabled. Accordingly, 86.4% of our cohort had favorable outcomes; 81.8% without disabilities (mRS 0) and 4.6% with minor disabilities (mRS 2). No complications related to the procedure itself were observed. Figure 1 represents the mRS changes between admission and discharge clinical state.

Low-grade bAVMs were mainly treated with surgery (50%) or embolization (16.7%) alone, while high-grade ones were mainly treated with radiosurgery (75%) or embolization (25%) only. Combined treatment was given to patients with low-grade bAVMs. A detailed description of applied interventions is listed in Table 3.

**Figure 2:**
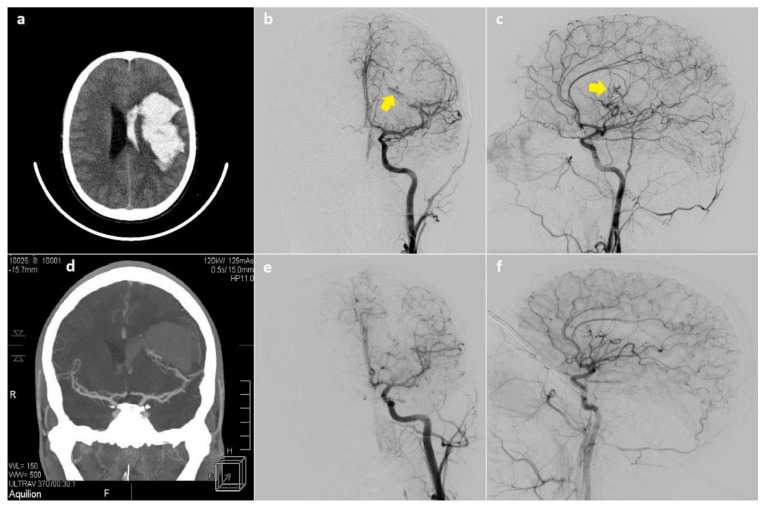
Imaging studies of the 16-year-old male patient. CT scan revealed intracerebral and intraventricular hemorrhage in the left hemisphere (**a**,**d**). Preoperative angiography demonstrated Spetzler-Martin III, arteriovenous malformation in the left temporal lobe (yellow arrows) (**b**,**c**). DSA examination performed 1.5 years after surgery showed complete excision of the malformation (**e**,**f**).

Posttreatment imaging data were available for 12 patients and showed complete bAVM obliteration among 5 surgically treated patients (Figure 2) and 2 patients who underwent embolization. The combined treatment strategies consisting of surgery with embolization and embolization with radiosurgery were less effective, since complete obliteration was achieved in 1 (50%) and 2 patients (66.7%), respectively.

## 4. Discussion

Bleeding from ruptured bAVMs constitutes the most prevalent cause of pediatric spontaneous intracranial hemorrhage, also representing the most common initial bAVM manifestation at admission [12,13]. The management of ruptured bAVMs should protect patients from rebleeding and subsequent significant morbidity. It has been shown that patients with the hemorrhagic presentation are at greater risk of rebleeding than those who were admitted with seizures (4.49 vs. 1.79% respectively) [14].

Several factors have been found to increase bleeding risk, including exclusive deep venous drainage, small nidus size, infratentorial location, female sex, and single draining vein [15,16,17]. Deep venous drainage has been identified by Ding et al. as the strongest independent predictor of bleeding [17]. These data suggest that small, compact bAVMs with a single deep draining vein are at the highest risk of bleeding. In our cohort, 91% of lesions were qualified as small (<3 cm), and 41% had deep venous drainage. We have not found lesions with nidus larger than 6 cm. The factors mentioned above constitute crucial elements on the Spetzler-Martin [9] and Lawton-Young Supplementary Grading Scale [18], which are routinely applied to assess surgical intervention risk.

Although the Spetzler-Martin [9] and Lawton-Young [18] scales facilitating the presurgical decision-making are well established, they have not been intended for predicting postinterventional outcomes exclusively in patients with ruptured bAVMs. Recently, several attempts have been made to develop a simple scale for predicting clinical outcomes in patients with ruptured bAVMs, regardless of the given treatment. In 2016, Neidert and colleagues established the first ruptured bAVMs-focused scale, named AVICH (AVM-related ICH) [19]. Recently, Silva et al. presented their Ruptured AVM Grading Scale (RAGS), which—according to authors’ findings—presented the highest predictive value among all previously published scales [20]. Although the RAGS scale is less detailed than AVICH, due to its simplicity, it has the potential to be implemented into daily clinical practice. However, it still lacks external validation; therefore, our study did not involve RAGS assessment. These predicting models would constitute a meaningful adjunct in the decision-making process, since the intervention’s expected benefit could be estimated more precisely.

Microsurgical resection, stereotactic radiosurgery, embolization, and various combinations of these methods constitute treatment options available at our institution for the management of ruptured pediatric bAVMs. As variable rates of morbidity, radiographic obliteration, and complications associated with each method have been reported in several retrospective series. A modality of choice in the event of ruptured bAVM in children should remain highly dependable on bAVM characteristics and the patient’s condition. However, the strict adherence to the Spetzler-Martin scale can help distinguish patients who may benefit from surgical intervention from those who should be managed with either endovascular therapy or radiosurgery; separately or as a combined treatment strategy.

Low-grade bAVMs (SM I–III) can be treated by microsurgical resection, with an acceptable risk of perioperative morbidity. Accordingly, this treatment approach, with or without prior endovascular embolization, remains the gold standard for definitive treatment of small accessible bAVMs in non-eloquent locations. In addition to immediate angiographic cure exceeding 80% [21], microsurgery provides definitive protection from rebleeding with a low risk of complications (0–12%) [22]. In our series, 9 patients with low-grade bAVMs (SM I–III) were treated with microsurgical resection as a standalone modality, with favorable outcomes (mRS 0–2) in 7 patients and complete obliteration in all patients whose postoperative angiographic data were available. In contrast, high-grade lesions (SM IV–V) carry a significant risk of surgery-related morbidity [9,18]. In our series, no patients with high-grade bAVMs underwent microsurgical excision of the lesion.

Endovascular embolization has gained a significant role in the multimodal treatment of bAVMs as a useful adjunct to other methods. Preoperative occlusion of deep, surgically inaccessible feeder arteries and intranidal aneurysms may facilitate subsequent microsurgical excision. Malformation volume reduction may also increase the effectiveness of the following radiosurgery. A complete obliteration rate of over 50% [23] was recently achieved, utilizing solely endovascular embolization in carefully selected lesions.

Stemer et al. [10] reported 21 embolizations in ruptured bAVMs, achieving complete occlusion in 52% of patients, 33% of who were cured with a single procedure. The procedure-related complication rate in this series was 10%. Hartmann et al. [24] reported new postembolization neurologic deficits in 14% of patients, including 2% with disabling deficits, most commonly due to ischemic strokes.

Although previous studies have shown that partial embolization—used to secure high-risk points in bAVM angioarchitecture before definitive treatment—may increase the risk of rebleeding [25], recent large retrospective studies concluded that any embolization (partial or complete) might enhance survival [26]. However, there is not enough substantive data to suggest that incomplete embolization reduces the risk of rebleeding during the latency interval before definitive microsurgical resection or radiosurgical obliteration. In our series, 3 patients who presented with ruptured low-grade bAVM (SM I–III) underwent embolization used as a solitary treatment modality, which enabled them to achieve complete obliteration of the lesion in 2 patients whose postoperative angiographic data was available. None of these 3 patients suffered from procedure-related complications, and all 3 were discharged with favorable clinical outcomes (mRS 0–2).

With the improvement of endovascular technology, embolization will continue to evolve and should be incorporated into the management of ruptured pediatric bAVMs, not only as an adjunctive modality, but also as a potentially curative method in some cases. In contrast to the transarterial approach, conventionally used in endovascular treatment of bAVMs, transvenous embolization has recently been proposed as a potentially curative intervention. This modality can be applied for carefully selected bAVMs, especially small (<3 cm), compact, ruptured lesions in deep-brain locations, with a single draining vein, lack of accessible arterial pedicles, and exclusive arterial supply by perforators [27,28]. Although the efficacy and safety of transvenous embolization require further evaluation, it might constitute a promising feasible alternative, providing curative obliteration of inaccessible nidal remnants after transarterial embolization, incomplete microsurgical excision, or failed stereotactic radiosurgery [27,28].

Low-grade but inaccessible bAVMs or those in eloquent locations might also be managed with stereotactic radiosurgery as a standalone modality, which, for appropriately selected lesions, provides an obliteration rate up to 81% with a 5% risk of complications [29]. However, radiosurgery-induced obliteration of bAVMs presents after a latency period of up to 3 years [30], during which the risk of rebleeding persists.

In our series, 6 patients with low-grade bAVMs (SM I–III) in supratentorial locations were managed, employing a multimodal approach. Complete obliteration of the lesion in postoperative DSA was confirmed in 2 of 3 patients who underwent partial embolization followed by radiosurgery, and in 1 of 2 patients managed utilizing microsurgical resection with prior partial embolization. Despite the inevitably higher procedural burden in patients who underwent multi-staged multimodal therapy, we did not observe any new neurologic deficits in this subgroup. Notably, we presume that lower obliteration rates observed in patients who underwent multimodal treatment do not point out a less effective therapeutic approach, but can be related to the greater complexity of lesions, which could not be completely obliterated by means of a single modality.

The best management option for high-grade bAVMs (SM grade IV–V) remains controversial. The involvement of deep or eloquent brain tissue by these lesions, combined with their greater size, often precludes safe and effective microsurgical resection.

Although the increased volume of high-grade lesions might contribute to lower radiosurgical obliteration rates [11], stereotactic radiosurgery should be considered in the management of surgically inaccessible bAVMs. Moreover, radiosurgery might constitute an effective modality for residual bAVMs after subtotal resection and in an attempt to lower surgical risks for bAVMs in functional brain locations [11].

In our series, 3 patients who presented with ruptured high-grade bAVMs (SM IV) in deep-brain locations underwent radiosurgical treatment, with favorable outcomes (mRS 0) and no apparent radiosurgery-related complications.

Careful patient qualification process tailored to specific bAVM characteristics facilitated favorable outcomes (mRS 0–2) in the majority (86.4%) of our patients and prevented procedure-related complications. We observed rebleeding neither in patients managed solely with radiosurgical treatment nor in patients treated with other modalities or their combinations. Neither mortality nor newly appearing deficits were observed after interventions. Three out of eight patients who initially presented with focal neurologic deficit remained moderately disabled (mRS 3), representing 13.6% of the entire cohort. Blauwbomme et al., in their long-term follow-up, observed 23.6% of unfavorable outcomes, with a 4.71% mortality rate [31]. In their retrospective study of 111 pediatric patients, Deng and colleagues found a 16.7% short-term disability rate and merely a 3.7% rate in the long-term follow-up [32].

### Limitations

Our study’s limitations include small sample size, incomplete posttreatment imaging data, and the lack of long-term mRS assessment. The retrospective study character carried a risk of patients’ selection bias for a particular treatment modality, which could have been avoided in the randomized or at least prospective study. The small sample size, with a considerable patients’ subdivision into many treatment modalities, precluded any statistical analysis of presented results. On the other hand, all patients have been followed up for the entire treatment process. Therefore all complications, including rebleeding incidents within the treatment period, have been precisely noted and stated in the manuscript.

## 5. Conclusions

Spontaneous hemorrhage resulting from bAVM rupture carries a significant risk of morbidity, which increases notably in the event of recurrent bleeding from these lesions; therefore, interventional prevention of rebleeding is highly endorsed. Employment of the Spetzler-Martin scale with adequate intervention selection resulted in an 86.4% rate of favorable postinterventional outcomes without any intervention-related morbidity. No patient experienced rebleeding during the entire observation period. Microsurgical resection of low-grade bAVMs remains an effective and safe definitive therapeutic approach. The management of high-grade bAVMs with radiosurgery or transarterial embolization can provide satisfactory outcomes without a high risk of disability.

## Figures and Tables

**Table 1:** Patients’ clinical presentation and clinical outcomes.

	No. of Patients (%)
Patients (*n*)	22
Male	11 (50)
Female	11 (50)
Age in years (mean, SD, range)	11.86 (range 2–17)
Presentation	
Altered level of consciousness	6 (27.3)
Seizures	3 (13.6)
Neurologic deficit	8 (36.4)
Headache	16 (72.7)
mRS at admission	
0	3 (13.6)
1	6 (27.3)
2	3 (13.6)
3	2 (9.1)
4	2 (9.1)
5	6 (27.3)
mRS at discharge	
0	18 (81.8)
1	0
2	1 (4.6)
3	3 (13.6)
4	0
5	0
Hunt-Hess score	
1	5 (22.7)
2	6 (27.3)
3	5 (22.7)
4	6 (27.3)
5	0

**Table 2:** Baseline radiological characteristics of brain arteriovenous malformations (bAVMs).

	No. of Patients (%)
Location	
Frontal	6 (27.3)
Fronto-parietal	1 (4.6)
Parietal	5 (22.7)
Occipital	4 (18.2)
Temporal	2 (9.1)
Deep	4 (18.2)
Laterality	
Right	11 (50)
Left	11 (50)
Hemorrhage	
ICH	20 (91)
IVH	11 (50)
SAH	1 (4.6)
Size of nidus	
0–3 cm	20 (91)
3–6 cm	2 (9.1)
>6 cm	0
Eloquent location	7 (31.8)
Deep venous drainage	9 (41)
Spetzler-Martin grade	
I	9 (41)
II	6 (27.3)
III	3 (13.6)
IV	4 (18.2)
V	0
Supplementary SM grade	
2	8 (36.4)
3	7 (31.8)
4	0
5	3 (13.6)
6	4 (18.2)
7	0
8	0
9	0
10	0

**Table 3:** Treatment strategy depending on the Spetzler-Martin grade.

	No. of Patients (%)	Surgery	Embolization	Radiosurgery	Embolization + Surgery	Embolization + Radiosurgery	Surgery + Radiosurgery
No. of patients	22	9	4	3	2	3	1
Low-grade AVMs							
I	9	6 (66.7)	1 (11.1)	0	0	2 (22.2)	0
II	6	2 (33.3)	2 (33.3)	0	0	1 (16.7)	1 (16.7)
III	3	1 (33.3)	0	0	2 (66.7)	0	0
High-grade AVMs							
IV	4	0	1 (25)	3 (75)	0	0	0
V	0	0	0	0	0	0	0

## Data Availability

The data generated during this study are available within the article. Datasets analyzed during the current study preparation are available from the corresponding author on reasonable request.
